# Maternal Transfer and Protective Role of the Alternative Complement Components in Zebrafish *Danio rerio*

**DOI:** 10.1371/journal.pone.0004498

**Published:** 2009-02-18

**Authors:** Zhiping Wang, Shicui Zhang, Zhou Tong, Lei Li, Guangfeng Wang

**Affiliations:** Department of Marine Biology, Ocean University of China, Qingdao, People's Republic of China; National Institute on Aging, United States of America

## Abstract

Embryos of most fish develop externally and are exposed to an aquatic environment full of potential pathogens, whereas they have little or only limited ability to mount an efficient and protective response. How fish embryos survive pathogenic attacks remains poorly defined. Here we demonstrate that the maternal immunization of female zebrafish with formalin-killed *Aeromonas hydrophila* causes a significant increase in C3 and Bf contents in the mother, a corresponding rise in the offspring, and induces a remarkable increase in the hemolytic activities in both the mother and offspring. In addition, the embryos derived from the immunized mother are significantly more tolerant to *A. hydrophila* challenge than those from the unimmunized fish, and blocking C3 and Bf activities by injection of the antibodies against C3 and Bf into the embryos render them more susceptible to *A. hydrophila*. These results clearly show that the protection of zebrafish embryos against *A. hydrophila* can be achieved by the maternally-transferred immunity of the complement system operating via the alternative pathway. This appears to be the first report providing *in vivo* evidences for the protective role of the alternative complement components in the early embryos of zebrafish, paving the way for insights into the *in vivo* function of other maternally-transferred factors in fish.

## Introduction

Eggs of most fish are released and fertilized externally, and the resulting embryos and larvae are therefore exposed to an aquatic environment full of potential pathogens capable of causing various types of diseases. During the early stages of development, fish embryos and larvae have little or only limited ability to synthesize immune-relevant molecules endogenously and their lymphoid organs are not yet fully matured [1], [2]. How they survive the pathogenic attacks in such a hostile environment is one of the key issues for reproductive and developmental immunology, however, information as such remains rudimentary to date.

Previous studies on several fish species have shown that maternal IgM is able to be transferred from mother to offspring [3]–[12]. Likewise, maternal transfer of innate immune factors including the complement component C3 [13]–[17], lectins [18]–[20], protease inhibitors [21], [22] and lysozymes [23], [24] to offspring has also been reported in different teleost species. Moreover, immunization of parents results in a significant increase in IgM levels [6], [11] and anti-protease and lysozyme activities [6] in their eggs compared to controls. These transferred maternal molecules have been proposed to be involved in the early defense against pathogens in developing fish embryos and larvae. For example, Wang et al. [16] have recently demonstrated by an *in vitro* assay system of complement activity that the the protection of early embryos of zebrafish *Danio rerio* against microbial attack can be attributed to maternal complement components operating via the alternative pathway (AP). However, it remains unknown whether these alternative complement components function *in vivo* during the early developmental stages. Therefore, the objectives of this study were to examine if the maternal alternative complement components are transferred from the immunized female *D. rerio* to offspring, and if so, to test if these components transferred affect the offspring immunity.

## Results

### Increase in C3 and Bf in immunized fish and eggs

**Figure 1:**
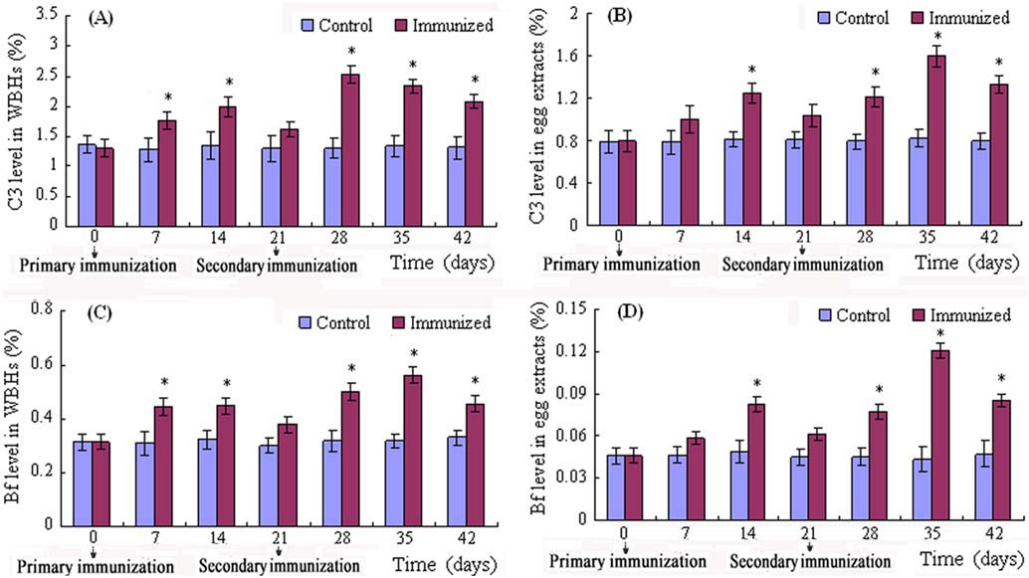
Influence of maternal immunization on C3 and Bf levels in the WBHs and egg extracts. Three immunized female *D. rerio* and three control females as well as their released eggs were sampled every 7 days after the primary immunization. Whole body homogenates (WBHs) and egg extracts were prepared, and used for the measurement of complement component levels. The complement component contents are presented as percentages of total protein of the WBHs and egg extracts. The symbol * represents a significant difference (*p*<0.05).

The protein contents of the whole body homogenates (WBHs) and egg extracts ranged from 13.1 to15.3 mg/ml, and from 15.7 to 18.6 mg/ml, respectively. Our previous study showed that the key components, complement component 3 (C3) and factor B (Bf), functioning in the AP, are present in the early embryos of *D. rerio*, and the complement system operating via the AP is largely attributable to the bacteriolytic activity in the early embryos, therefore the contents of C3 and Bf were determined and compared between the WBHs and egg extracts. Injection of PBS into female *D. rerio* resulted in little changes in C3 and Bf contents in both the WBHs and egg extracts (**Fig. 1**). In contrast, the immunization with formalin-killed *Aeromonas hydrophila* induced a significant increase in C3 and Bf levels in both the WBHs and egg extracts. The C3 and Bf levels in the WBHs peaked at week 2 and week 1, respectively, following the primary immunization; they both decreased slightly and then increased significantly after the secondary immunization (**Fig. 1A and C**). Interestingly, the maternal immunization also caused a marked corresponding rise in C3 and Bf levels in the egg extracts, and the fluctuation profile of the C3 and Bf contents in the egg extracts generally coincided with that in the WBHs (**Fig. 1B and D**). This indicated that the maternal immunization induced a significant increase of C3 and Bf levels in the mothers, which in turn resulted in a marked increase in C3 and Bf levels in their eggs. Moreover, the statistical analysis revealed a significant difference between the peak values of C3 and Bf amounts in both the WBHs and egg extracts after the primary and secondary immunizations, suggesting that the secondary immunization caused a significant increase in these factors in the mothers as well as in the offspring.

### Increase in hemolytic activity in immunized fish and eggs

**Figure 2:**
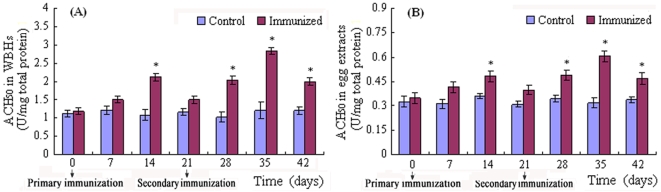
Influence of maternal immunization on the ACH_50_ in the WBHs and egg extracts. Three immunized female *D. rerio* and three control females as well as their released eggs were sampled every 7 days after the primary immunization. WBHs and egg extracts were prepared, and used for the measurement of hemolytic activities. The symbol * represents a significant difference (*p*<0.05) between the time-points indicated and day 0 post the primary immunization.

The haemolytic activity driven by the AP was assayed, and the reciprocal of the WBH or egg extract dilution causing 50% lysis of the rabbit red blood cells (RaRBC) was designated as the ACH_50_. Injection with PBS did not exert any influence on ACH_50_ in both the WBHs and egg extracts, while the maternal immunization resulted in a marked increase in ACH_50_ in both the WBHs and egg extracts (**Fig. 2A and B**). Notably, the change in ACH_50_ in the egg extracts had a fluctuation profile resembling exactly that observed in the WBHs. Specifically, the ACH_50_ in both the WBHs and egg extracts increased moderately after the first week, peaked after the second week, and decreased to the control level after the third week post primary immunization; following the secondary immunization, the ACH_50_ in both the WBHs and egg extracts increased significantly at the first week time point (fourth week since the primary immunization), peaked at the second week time point (fifth week since the primary immunization), and remained significantly higher after three weeks (sixth week since the primary immunization). This suggested that, the innate immunity exemplified by the hemolytic activity in the immunized mothers was also transferred to their offspring.

**Figure 3:**
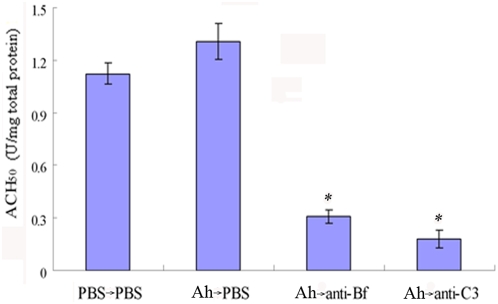
Effects of anti-C3 and anti-Bf antibodies on the hemolytic activity. The fish were injected first with the formalin-killed *A. hydrophila*, and , 6 days later with anti-C3 antibodies and anti-Bf antibodies, respectively.. The WBHs were prepared at 24 h after the injection of anti-C3 antibody or anti-Bf antibody, and the ACH50 was examined. Ah: *A. hydrophila*.

To correlate the increased hemolytic activity with elevated values of C3 and Bf, female *D. rerio* were injected first with formalin-killed *A. hydrophila*, and then 6 days later with anti-C3 antibody or anti-Bf antibody to inactivate the complement component. The fish were sampled 24 h after the injection of anti-C3 antibodies or anti-Bf antibodies, the WBHs were prepared and the ACH_50_ was measured. As expected (see **Fig. 2** above), the ACH_50_ in the WBHs was slightly increased by immunizing *D. rerio* with *A. hydrophila* when compared to the control (PBS-injected group), but the ACH_50_ was remarkably reduced by the pre-injection of anti-C3 and anti-Bf antibodies (**Fig. 3**). This provided additional support for the previous observations that the hemolytic activity was correlated with the levels of complement components [30]–[32].

### Increase in anti-infection activity in immunized eggs

**Figure 4:**
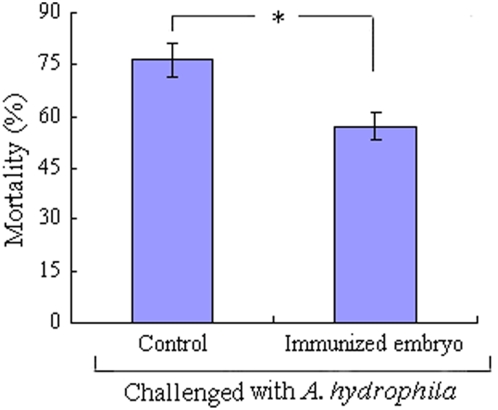
Influence of maternal immunization on the anti-infection activity of *D. rerio* embryos. The embryos from the immunized and control females were both challenged by injection of live *A. hydrophila* 24–28 h post fertilization, and the cumulative mortalities at 24 h after injection were counted. The symbol * represents a significant difference (*p*<0.05) between the time-points indicated and day 0 post the primary immunization.

**Fig. 4** shows the cumulative percent mortalities of the embryos obtained from both the unimmunized and immunized mother fish at 24 h after the injection with live *A. hydrophila*. The percent mortality of the embryos from the immunized *D. rerio* was approximately 57%, whereas the mortality of the embryos from the unimmunized *D. rerio* was about 76%. The mortality of the embryos from the immunized mother was significantly lower than that of the embryos from the unimmunized mother (*p*<0.05), suggesting that the maternal immunization rendered the offspring more tolerant to the challenge with *A. hydrophila*.

**Figure 5:**
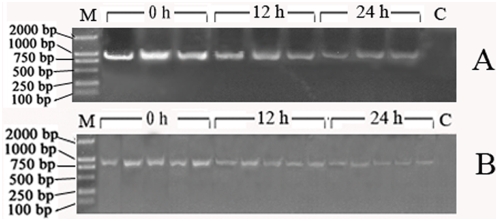
PCR analysis of *A. hydrophila* 16S rRNA gene in the embryos. (A) A total of 10 embryos were collected each time at 0, 12 and 24 h post the bacterial injection, respectively, and DNAs were isolated and used to amplify the specific region of *A. hydrophila* 16S rRNA gene. (B) A single embryo was collected each time at 0, 12 and 24 h post the bacterial injection, respectively, and DNAs were isolated and used to amplify the specific region of *A. hydrophila* 16S rRNA gene. The PCR products were electrophoresed in 1% agarose and the bands were recorded using the gel imaging system. M: Marker; C: Control.

To further verify the killing of live *A. hydrophila* by the embryos, PCR analysis was performed using a primer set (sense primer, 5′- AATACCGCATACGCCCTAC-3′; anti-sense primer, 5′- AACCCAACATCTCACGACAC-3′) amplifying a specific region of *A. hydrophila* 16S rRNA gene. As shown in **Fig. 5**, no band was observed in the control sample, but intense bands were seen in the embryos collected soon after the bacterial injection (0 h), and the band intensities apparently decreased with time (at 12 h and 24 h), suggesting the lysis of the bacterium by the embryos.

### Involvement of C3 and Bf in anti-infection activity in eggs

**Figure 6:**
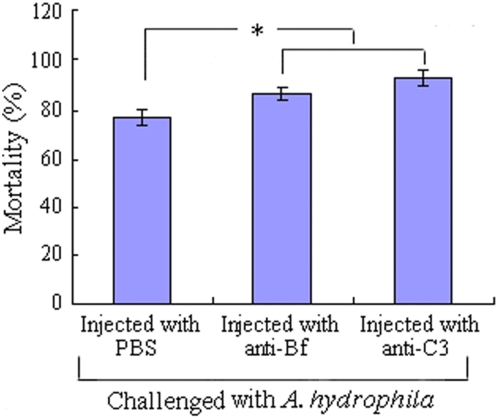
Influence of anti-C3 and anti-Bf antibodies on the anti-infection activity of *D. rerio* embryos. The embryos were first microinjected at 24–28 h post fertilization with antibodies against C3 or Bf, and then challenged by injection of live *A. hydrophila* 1 h later. The cumulative mortalities were counted at 24 h following the bacterial injection. The symbol * means a significant difference (*p*<0.05).

To test if the key components of the AP, C3 and Bf, are involved in the protection of the embryos against the bacterial attack *in vivo*, antibodies against C3 and Bf were microinjectedinto the embryos to block C3 and Bf actions, respectively. It was found that the microinjection of the antibodies against C3 and Bf both resulted in a significant increase in the mortality of the embryos challenged with live *A. hydrophila*, with cumulative mortalities of ∼94% and ∼87%, respectively, contrasting to that of ∼77% in control (**Fig. 6**). It was clear that both C3 and Bf were involved in the antibacterial activity in the developing embryos, indicating that the complement system operating via the AP was one of the most important factors associated with the protection of the embryos *in vivo*.

## Discussion

Due to lack of immune competence at the early stages, how the piscine embryos survive pathogenic attacks remains poorly defined. Previously, passive transfer of both IgM and IgM-related immunity into offspring by immunizing mother has been reported in several teleostean fish including tilapia [25], [26], rainbow trout [27], salmon [28], sea bream [29], and carp [11]. In contrast, similar studies focussing on innate immune factors are much more limited. In this study, we first demonstrate that the immunization of female *D. rerio* with the formalin-killed *A. hydrophila* results in a significant increase in C3 and Bf contents in the mother and a corresponding rise in the offspring (**Fig. 1**), confirming the presence of C3 and Bf in the eggs of *D. rerio* as reported by Wang et al. [16]. To test if the innate immunity bestowed by C3 and Bf is transferred, we determined the hemolytic activities exemplified by ACH_50_ in the WBHs and egg extracts, which correlate well with the protein levels of complement contents [30]–[32]. It was found that the maternal immunization also causes a remarkable increase in the hemolytic activity in the mother and a corresponding increase in the offspring (**Figs. 2** **and** **3**). Furthermore, the developing embryos derived from the immunized *D. rerio* are significantly more tolerant than those from the unimmunized mother upon challenge with *A. hydrophila* (**Figs. 4** **and** **5**). Moreover, abrogation of C3 and Bf activities by injection of the antibodies against C3 and Bf into the developing embryos to block the AP, followed by the challenge with *A. hydrophila*, leads to a significantly increased mortality in the target embryos (**Fig. 6**). Taken together, these data clearly show that not only C3 and Bf are able to be transferred from the immunized female *D. rerio* to offspring, but these factors are also able to confer protection to the offspring as well. This appears the first report elucidating the protective role *in vivo* of maternally-transferred alternative complement components in the developing embryos of non-mammalian vertebrates.

Complement is a sophisticated proteolysis system which plays two major roles in humoral immunity: one is to lyse pathogenic cells directly; and the other is to opsonize pathogens for phagocytosis and to recruit immunocytes to the reaction focus. The present study clearly shows the presence of C3 and Bf inside the eggs/embryos. As the macrophages are not observed in *D. rerio* embryos younger than 2-day-old larvae [33], [34], the key components C3 and Bf functioning in the AP may exert their antibacterial activity by interacting with and destabilizing the invading pathogens, leading to the pathogenic cell lysis (**Fig. 5**). In agreement, the maternally transferred IgM stored in the yolk sac in *D. rerio* embryos is also able to retain the capability to bind antigens [3], [25], [26], [29]. When the macrophages occur later in the embryos, C3 and Bf may also function in opsonizing the pathogenic cells for phagocytosis.

It is of note that after the secondary immunization, both C3 and Bf contents are significantly elevated in the mother and offspring (**Fig. 1**). Similarly, the hemolytic activities are also markedly enhanced in the mother and offspring following the secondary immunization (**Fig. 2**). These suggest that repeated maternal immunizations are able to strengthen the maternal immunity of the offspring, which can be potentially applied to protect fish too young to mount an immune response of their own. Additional studies will be required, however, to determine if this transferred immunity can be maintained for an extended period of time.

To summarize, we have shown for the first time that the *in vivo* protection of zebrafish embryos against *A. hydrophila* can be achieved by the maternally-transferred immunity of the complement system operating via the AP. As the maternal transfer of complement components to piscine offspring appears widespread, the complement-mediated maternal immunity may be a general route for protecting fish embryos and larvae from pathogenic attacks. It will be of interest in the future to study if the maternal IgM transferred can aid activation of the AP in the developing fish embryos.

## Materials and Methods

### Reagents and solutions

Ethylenediaminetetraacetic acid (EDTA), ethyleneglycol-bis (B-aminoethyl ether)-N, N, N′-tetraacetic acid (EGTA), bovine serum albumin (BSA), gelatin, *O*-phenylenediamine dihydrochloride (OPD), 3-aminobenzoic acid methyl ester (MS222), dichloro-diphenyl-trichloroethane (DDT) and Triton X-100 were purchased from Sigma (USA). Tryptic soy broth (TSB) was procured from OXOID (UK), and complement C3 and Bf standards were from ADL (USA), rabbit anti-human C3 antibody from Abcam (UK), goat anti-human Bf antibody from R&D (USA), and horseradish peroxidase (HRP)-labeled rabbit anti-goat IgG and goat anti-rabbit IgG from Boster (China). All the other chemicals used were analytical reagents.

The buffers used in the experiment for the alternative complement activity (ACH_50_) assay were: gelatin verona1 buffer (GVB), isotonic veronal-buffered saline (pH 7.3) containing 5 mM sodium bartiturate, 446 mM NaCl and 0.1% gelatin; EDTA-GVB, GVB containing 10 mM EDTA; and Mg^2+^-EGTA-GVB, GVB containing 10 mM Mg^2+^ and 10 mM EGTA (Sunyer and Tort, 1995).

### Preparation of bacterium *Aeromonas hydrophila*

The bacterium *Aeromonas hydrophila* LSA 20, pathogenic to *D. rerio*, was a gift of Dr. Z. L. Mo in the Institute of Oceanology, Chinese Academy of Sciences. It was grown at 28°C in TSB for 16 h. After enumerating with a blood cell counter, the bacterial cells were washed three times with sterile PBS, re-suspended at a density of 1.33×10^8^ cells/ml, and used for the bacterial challenge experiments. Also, the bacterial cells were inactivated by the fixation with 0.4% formalin at 28°C for 24 h, harvested by centrifugation at 3000 g for 10 min at 4°C, re-suspended in PBS at 2.5×10^9^ cells/ml, and used for the immunization experiments.

### Preparation of rabbit red blood cells (RaRBC)

Blood was collected from the ear artery of a New Zealand male rabbit, mixed immediately with an equal volume of Alsever's solution and centrifuged at 500 g for 5 min. The pelleted RaRBCs were washed three times with Mg^2+^-EGTA-GVB, adjusted to 2.5×10^8^ cells/ml with the same buffer, stored at 4°C and used within a week [35].

### Fish immunization, sample collection and preparation

**Figure 7:**
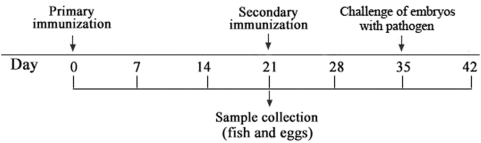
A schematic diagram of the experiment.

In total, 60 sexually-mature female *D. rerio* at age of 6 months old were divided into two groups. Fish of the experimental group were anaesthetized with Tris-buffered MS222 (168 µg/ml), injected intramuscularly with 20 µl of *A. hydrophila* suspension, and followed by a secondary immunization after 3 weeks to strengthen the immune effects as shown in **Fig. 7**. Similarly, fish of the control group were injected with 20 µl of sterile PBS. Immunized and control (unimmunized) fish were maintained in separate tanks with well-aerated tap water at 26±1°C.

Immunized and control female fish were mated with normal male *D. rereio* at a female to male ratio of 2∶1 prior to the primary immunization and every 7 days post the primary immunization (zebrafish reproduce eggs weekly in appropriate conditions). Naturally fertilized eggs, which were usually at 4- to 8-cell stage, were collected, washed three times with sterile PBS, and homogenized. The homogenates were centrifuged at 15 000 g for 30 min at 4°C, and the supernatants called egg extracts were pooled and stored at −70°C until used. For the fertilized eggs collected on the 35^th^ days post the primary immunization (i.e., at the second week post the secondary immunization), about 100 eggs were taken from each group, and cultured for the following bacterial challenge experiment.

Immediately after spawning, three female *D. rerio* were each sampled prior to the primary immunization and every 7 days post the primary immunization. As the serum is rather difficult to collect from zebrafish because of its small size, the whole body homogenates (WBHs) were therefore prepared by the method of Holbech et al. [36] and used to measure the complement component levels and hemolytic activity. The fish sampled were crushed in a mortar filled with liquid nitrogen, mixed with 2 volume of the body weight of ice-cold PBS (w/v), and centrifuged at 15 000 g for 30 min at 4°C. The supernatants were pooled and stored at −70°C until used.

Another 24 female *D. rerio* were divided into 4 groups, A, B, C and D. The fish in groups A, B and C were anaesthetized and injected with 20 µl of *A. hydrophila* suspension at 2.5×10^9^ cells/ml, and the fish in group D were injected with the same volume of sterile PBS as control. Six days later, the fish in groups A and B were injected with 20 µl of anti-C3 antibody solution at 100 mg/ml or 20 µl of anti-Bf antibody solution at 100 mg/ml, and the fish in groups C and D injected with 20 µl of sterile PBS. The fish in each group were collected 24 h later, and the WBHs were prepared as described above.

The protein concentrations were determined by the method of Bradford [37] with BSA as standard.

### Enzyme-inked immunosorbent assay (ELISA)

Our previous work [16] has shown that the rabbit anti-human C3 antibody and goat anti-human Bf antibody can react with zebrafish C3 and Bf, respectively, therefore, these antibodies were selected to measure the contents of C3 and Bf in the WBHs and egg extracts by ELISA. In brief, the wells in 96-well microtiter plates (Costar) were each coated with 100 µl of C3 and Bf standards, WHBs, and egg extracts, respectively, and placed at 4°C overnight. After washing 5 times with PBST (PBS containg 0.1% Tween-20), the wells were each blocked with 100 µl of 3% BSA at 32°C for 1 h, followed by addition of 100 µl of rabbit anti-human C3 antibody diluted at 1∶1000 with PBS or goat anti-human Bf antibody diluted at 1∶400 with PBS into each well and incubation at 32°C for 1 h. The wells were washed with PBST, added with 100 µl of HRP-labeled anti-rabbit IgG diluted at 1∶1000 with PBS or anti-goat IgG diluted at 1∶1000 with PBS, and incubated at 37°C for 1 h. Subsequently, an aliquot of 75 µl of 0.4 mg/ml OPD in 51.4 mM Na_2_HPO_4_, 24.3 mM citric acid and 0.045‰ H_2_O_2_ (pH 5.0) was added to each well, and incubated at 37°C for 20 min in dark. To terminate the reactions, 25 µl of 2 M H_2_SO_4_ was added into each well, and absorbance at 492 nm was monitored by a microplate reader (GENios Plus, Tecan).

### Hemolytic activity assay

The haemolytic activity driven by the AP was assayed as described by Sunyer and Tort [38]. The assay was carried out in Eppendorff tubes. A volume of 25 µl of RaRBC suspension was mixed with 100 µl of WBHs or egg extracts that were both diluted 2-fold serially in Mg^2+^-EGTA-GVB. The tubes were incubated at 25°C for 100 min, with gentle shaking. The reactions were stopped by addition of 1 ml of cold EDTA-GVB. The tubes were then centrifuged at 1600 g for 5 min and the extent of haemolysis was determined by measuring the OD of the supernatants at 414 nm. Total (100%) hemolysis was given by the optical reading of the supernatant obtained by centrifuging the mixture of 25 µl of the RaRBC suspension plus 1100 µl of distilled water. The reciprocal of the WBH or egg extract dilution causing 50% lysis of the RaRBC was designated as the ACH_50_. The results were normalized by the protein contents of the WBHs or egg extracts and presented as U/mg.

### Bacterial challenge assay

To test if the embryos derived from the immunized females were more resistant to bacterial challenge, the pharyngula stage embryos (24 to 28 h post fertilization) developed from the fertilized eggs collected from the immunized and control females on the 35^th^ days post the primary immunization were dechorionated, anaesthetized with 0.02% MS222 and microinjected in the yolk sac with ∼6 nl (∼400 cells) of live *A. hydrophila* suspension. The cumulative mortalities were counted and calculated at 24 h after the bacterial injection.

To verify the killing of *A. hydrophila* by the embryos, another 100 healthy embryos derived from the control females were dechorionated, and microinjected with live *A. hydrophila* as described above. A total of 10 embryos as well as a single embryo were collected each time at 0, 12 and 24 h post the bacterial injection, respectively. Normal (untreated) embryos were also collected to serve as controls. DNAs were isolated from the embryos according to the method of Li et al. [39]. Briefly, the embryos were washed 3 times with sterile H_2_O, lysed with 50 µl of sterile alkaline embryo lysis (200 mmol/L NaOH, 50 mmol/L DDT and 1% Triton X-100), incubated at 65–70°C for 20 min and then cooled on ice immediately. An aliquot of 3 µl of the lysates was sampled and used as DNA template. The PCR was carried out under the following conditions: 95°C for 10 min, followed by 38 cycles of 95°C for 30 s, 55.5°C for 30 s, and 72°C for 1 min and one cycle of 72°C for 7 min. The PCR primers used were the sense primer 5′- AATACCGCATACGCCCTAC-3′ and anti-sense primer 5′- AACCCAACATCTCACGACAC-3′, which were designed on the basis of *A. hydrophila* 16S rRNA sequence (GenBank accession no. DQ207728) and were able to amplify a specific region of *A. hydrophila* 16S rRNA gene.

To assay if the complement system operating via the AP is associated with the protection *in vivo* of the embryos, 300 pharyngula stage embryos derived from the non-immunized females were dechorionated, anaesthetized, and divided into three groups A, B and C. The embryos in group A were individually microinjected in the yolk sac with ∼6 nl of rabbit anti-human C3 antibody (∼0.28 ng), the embryos in group B were each injected with ∼6 nl of goat anti-human Bf antibody (∼0.24 ng), and the embryos in group C injected with ∼6 nl of sterile PBS (control). All the embryos in the three groups were then each microinjected in the yolk sac with ∼6 nl of live *A. hydrophila* (∼400 cells) at 1 h later. The cumulative mortalities were accounted at 24 h after the bacterial injection.

### Statistical analysis

All experiments were performed in triplicate, and repeated at least three times. Data were subjected to statistical evaluation with one-way analysis of variance, and difference at *p*<0.05 was considered significant. All data were expressed as mean±standard deviation (SD).
